# Who has a beef with reducing red and processed meat consumption? A media framing analysis

**DOI:** 10.1017/S1368980021004092

**Published:** 2022-03

**Authors:** Katherine Sievert, Mark Lawrence, Christine Parker, Cherie A Russell, Phillip Baker

**Affiliations:** 1 School of Exercise and Nutrition Sciences, Deakin University, Burwood Highway, Burwood, VIC 3125, Australia; 2 Institute for Physical Activity and Nutrition, Deakin University, Geelong, Australia; 3 Melbourne Law School, The University of Melbourne, Melbourne, Australia

**Keywords:** Meat reduction, Framing, Media, Sustainable food systems, Health, Environment, Vegan agenda

## Abstract

**Objective:**

Diets high in red and processed meat (RPM) contribute substantially to environmental degradation, greenhouse gas emissions and the global burden of chronic disease. High-profile reports have called for significant global RPM reduction, especially in high-income settings. Despite this, policy attention and political priority for the issue are low.

**Design:**

The study used a theoretically guided framing analysis to identify frames used by various interest groups in relation to reducing RPM in online news media articles published in the months around the release of four high-profile reports by authoritative organisations that included a focus on the impacts of high RPM production and/or consumption.

**Setting:**

Four major RPM producing and consuming countries – USA, United Kingdom, Australia and New Zealand.

**Participants:**

None.

**Results:**

Hundred and fifty news media articles were included. Articles reported the views of academics, policymakers, industry representatives and the article authors themselves. RPM reduction was remarkably polarising. Industry frequently framed RPM reduction as part of a ‘Vegan Agenda’ or as advocated by an elite minority. Reducing RPM was also depicted as an infringement on personal choice and traditional values. Many interest groups attempted to discredit the reports by citing a lack of consensus on the evidence, or that only certain forms of farming and processing were harmful. Academics and nutrition experts were more likely to be cited in articles that were aligned with the findings of the reports.

**Conclusions:**

The polarisation of RPM reduction has led to a binary conflict between pro- and anti-meat reduction actors. This division may diminish the extent to which political leaders will prioritise this in policy agendas. Using nuanced and context-dependent messaging could ensure the narratives around meat are less conflicting and more effective in addressing health and environmental harms associated with RPM.

Industrial food systems are among the leading contributors to the increased health and environmental burdens experienced by human populations worldwide^(1,2)^. Excessive production and consumption of both animal-source and ultra-processed foods are major harms that deserve urgent policy attention^(3,4)^. Red and processed meats (RPM) are overrepresented in diets of high-income populations, and increasingly, those of lower- and middle-income (LMIC) diets^(5,6)^. Although red meat is a recognised source of key minerals and vitamins^(7)^, such as vitamin B_12_, and can have a place in a healthy diet, excessive consumption is associated with significant health risks, including cancer^(8)^. Processed meats are classified as a Category 1 carcinogen by the WHO^(9)^ and have been associated with increased risk for colorectal cancer^(10)^, with emerging evidence of their association with stomach cancer^(11)^. The growing proportion of RPM in diets can also displace whole grains, fruits and vegetables^(1)^, further contributing to the global burden of disease^(1)^. In addition, the industrial production of RPM generates significant environmental harms, particularly from intensive farming practices that rely on grain-feed. Industrial production of RPM involves often-global supply chains that require resource-intensive inputs (such as feed crop production), which exceed planetary boundaries for greenhouse gas emissions, deforestation, freshwater use and land conservation^(12,13)^. Intensive agriculture, which in countries like the US comprises the majority of RPM production, also harms animal welfare^(14)^ and is a leading contributor to anti-microbial resistance^(15)^, as well as increased frequency of novel zoonotic virus outbreaks, like the current COVID-19 pandemic^(16)^.

Recognising these concerns, many global authoritative organisations are calling for a ‘food systems transformation’^(2,17,18)^. High-profile reports such as the EAT-Lancet Commission for Healthy and Sustainable Food Systems and the Intergovernmental Panel on Climate Change (IPCC) on Climate Change and Land have called for significant reductions in total global meat production and consumption – particularly in higher-income countries – as a fundamental action towards achieving healthy and sustainable food systems^(2)^. Suggested interventions to accomplish this have included suites of policy actions that directly target both the design and function of food systems (such as governance and accountability mechanisms, as well as food environment design), as well as interventions that concentrate on shaping specific feedback loops within production processes that, in conjunction with other interventions, influence the whole system (e.g. emissions taxes or labelling schemes).

Despite these calls for transformation, policy attention and political priority for the notion of RPM reduction – what we define as *systematic efforts, involving actions throughout the food system, to reduce the production, marketing and consumption of red and processed meat* ^(19)^ – appears to be low. Few (if any) governments have undertaken systematic policy action on RPM reduction. A number of factors contribute to this policy inertia, including contention as to whether RPM is actually a problem^(20)^ and politically entrenched opposition due to the cultural, historical and economic importance of meat^(21,22)^. There is a lack of academic attention to the power of ideas and narratives surrounding RPM, and how these are reinforced and employed by various interest groups to promote or resist its reduction^(19)^. This paucity is an oversight, as the way issues are framed in civic discourses can have a significant influence on the knowledge, attitudes and behaviours of the general public^(23)^.

Ideas can pervade and perpetuate high levels of RPM consumption. Such ideas can be established, reinforced or manipulated by different actors to further their interests or counteract their opponents. This is referred to as ‘discursive power’ – the use of ideas to generate attention, shape-scientific evidence, frame debates and influence social norms^(24,25)^. A growing body of literature on the ‘commercial determinants of health’ (CDoH) shows how corporations and industry groups use diverse practices – both market (e.g. pricing strategies, advertising and promotion) and political (e.g. lobbying, corporate research and voluntary self-regulation) – to promote and sustain markets for products harmful to health^(26)^, including, for example, tobacco, alcohol and ultra-processed foods^(3,27)^. Yet few studies have examined the RPM industry, including its framing strategies. Previous studies on how meat is represented in the media have focused on consumer awareness of the environmental^(28)^ or health impacts^(29)^, perceptions of information trustworthiness^(30)^, levels of media coverage on meat^(31)^, and the influence of media on consumer purchasing habits^(32)^. One study explored the changing narratives of meat in response to public crises, such as the BSE crisis, and found that varying interest groups – including the meat industry and vegan movements – contributed to a strongly polarised discourse^(33)^. However, this study only collected articles from one media outlet and did not attribute frames to specific actors.

The aim of this study is to understand how the challenge of RPM reduction is represented and interpreted in news media. The objective is to identify frames used by different interest groups in news media in response to RPM reduction recommendations by high-profile reports on healthy and sustainable diets and food systems transformation. Because the RPM reduction recommendations in these reports could be perceived as either a threat (e.g. to the meat industry or consumers) or an opportunity (to health and environment experts or alternative protein industries) by different groups, we expect them to generate significant contestation in news coverage. We ask how is meat reduction framed and contested by different interest groups? What implications do these framings and contestations have for future actions towards achieving a food systems transformation?

## Methods

### Research design

We adopted a qualitative, theoretically guided framing analysis method^(34,35)^. This involved three steps: (i) document collection; (ii) thematic analysis and (iii) synthesis of final themes and results.

For feasibility, the scope of this study was limited to four major RPM producing and consuming countries – USA, United Kingdom, Australia and New Zealand, with English as the primary language spoken. Online news media outlets in these countries that published articles 2 months prior, and 4 months following, the release of one of four high-profile reports containing RPM reduction recommendations were included in this study: (1) Livestock’s Long Shadow (November 2006), FAO of the UN, Rome, Italy^(36)^; (2) Monographs on the Evaluation of Carcinogenic Risks to Humans – Red meat and processed meat (October 2015), WHO – International Agency for Research on Cancer (WHO-IARC), Lyon, France^(37)^; (3) Food in the Anthropocene: Healthy Diets from Sustainable Food Systems. As part of the EAT-Lancet Commission on Food, Planet, Health (January 2019), EAT Foundation, online^(2)^ (heretofore EAT-Lancet); and (4) Climate Change and Land (August 2019), Intergovernmental Panel on Climate Change, Geneva, Switzerland^(38)^ (heretofore IPCC).

Each report included a focus on the impact of high RPM consumption and/or production on human health and/or the environment. Whilst many reports have been published since 2000 that focus on the harms associated with RPM, these four reports generated substantial media attention. A table of their specific conclusions or recommendations for RPM reduction is included as Supplemental Text 1.

### Theory

In order to understand framing and contestation of RPM reduction in news media, we adopted a constructivist method^(39,40)^. Constructivism is an approach to understanding the nature of reality as ‘constructed’ by human beings, in contrast to the ‘materialist’ nature of reality that is measurable/observable and exists independent of human interpretation^(41)^. A constructivist approach considers the role of norms, ideas, knowledge and culture in a given social or political environment, with an emphasis on inter-subjective or widely held views, that subsequently shape the ‘interests and identities of purposive actors’^(40,42)^. These are usually offered in the form of a ‘frame’ – a central unifying idea, or set of ideas, used to interpret reality, and convey meaning^(43–44)^. Frames are inescapable features of mass communication that are relied upon as a means of interpreting the political and social worlds; however, they are frequently applied with intention and strategy by actors in an attempt to persuade or garner support for a specific agenda or policy movement^(45,46)^.

‘Policy frames’ are ideas that help define what is, and what is not, considered to be a ‘policy problem’. They also inform who is, and who is not, considered to be a legitimate stakeholder in the policy process, and what forms of knowledge and types of evidence are relevant and considered acceptable^(45,47,48)^. Policy scholars have documented several ways in which different actors construct and utilise policy frames to further their agenda or goal. These include defining the problems (or the very recognition of something *being* a problem); determining causality or causal actors; assigning responsibility for solutions; and positioning the issue for tractability and benefit, in order to assemble support or counter opposition^(49,50)^. Frames gain traction when supported by a number of actors, such as interest groups or academic experts with shared values and beliefs – referred to as ‘coordinative discourses’^(42)^.

The resonance of frames can be either reinforced or diminished within the pre-existing paradigms and deeper ideologies that guide policy-decision making. A ‘paradigm’ comprises a coherent set of ideas that, in a policy context, not only contribute to the setting of policy targets but also the ‘best’ method to achieve those targets, as well as the very nature of the problem the policy is aiming to address^(43,51)^. These sets of ideas can become ‘institutionalised’ as formal laws and regulations, but also in the informal norms and beliefs that guide decision-making. Such institutions can then work as ideational filters – selecting out which ideas are, and are not, deemed acceptable for consideration. In the context of RPM and food more generally, ‘productivism’ is a deeply entrenched policy paradigm in many Western countries, including the USA, the UK and Australia. It orientates policymaking towards the goals of efficiency, market expansion and trade^(52)^ and thus supports high levels of RPM production. Productivism is embedded within a deeper ideology, neoliberalism, which emphasises small government, free markets and devolved governance, including emphasis on more private sector involvement in policy and an expanded role for individual consumer responsibility^(53,54)^.

More broadly, ideologies such as ‘carnism’ help underpin and uphold these policy goals. Carnism contends that meat is a ‘natural’, ‘normal’ and ‘necessary’ feature of the human diet^(22)^. While meat has been a feature of human diets worldwide for millennia and contributes a highly bioavailable source of vitamins and nutrients, it is now being consumed at levels higher than nutritionally necessary – in high-income countries especially – and constitutes a larger proportion of these populations’ overall diet, relative to fruit and vegetable intake^(55)^. Arcari describes it as an embedded value in many societies globally, one that normalises excessive meat consumption and in turn justifying modern intensive agriculture practices^(21)^. As carnism helps reinforce the ‘need’ for such high levels of meat intake, it simultaneously obscures the associated harms to human, animal and planetary health that that amount of meat consumption contributes^(21)^.

The use of evidence when forming policy priorities can also be subject to wide variability. In contrast to ‘ideas’, which carry a sense of social construct, ‘evidence’ is assumed to be objective and free of political bias. Policymaking should be underscored by the best available evidence at a given time; however, many policies are heavily influenced by a variety of other factors (such as ideas) and can even be developed counter to the best available scientific knowledge^(48)^. Furthermore, evidence can be selectively generated, synthesised and translated to support one particular paradigm and/or obscure a competing one^(56)^. It is crucial to understand how proposed policy objectives (such as RPM reduction) become subject to diverging interpretation by different actors with different interests^(48)^.

To ascertain *which* ideas are being interpreted and communicated (i.e. framed) in social discourses, including news media, a framing analysis is often the method of choice. It is important to understand what frames are being conveyed by various interest groups across news outlets and networks, and how the ideas conveyed are received and prioritised by the general public, and as well as in policy agendas^(50,57)^.

### Positionality statement

How the authors interpret and portray RPM reduction can influence this research^(58)^, with the potential to influence all components of the study. The authors acknowledge that RPM (as well as animal-source foods) can, and does, play an important role in health and nutrition for many people. There are currently many individuals who lack access to adequate food of any type, and the burden of RPM reduction should not fall on them. We acknowledge that animals can be, and are used, in ecologically sensitive ways in some traditional and modern farming systems, and that the foods produced from animals are an important part of most food cultures and cuisines. Our concern in this paper is with RPM production and consumption levels well in excess of human need and planetary boundaries, and frequently at the expense of animal welfare.

### Document collection

A structured search of the ProQuest International Newsstream database^(59)^ was conducted in July 2020 for 6-month time frames surrounding publication of the four reports. This database was selected as it contains archives from over 2800 of the world’s top news sources over several decades^(60)^ and was deemed to contain a wide breadth of news media sources with a range of political leanings. In order to determine relevant search terms, PDF of each report and the accompanying policy and media summaries were uploaded to qualitative analysis software NVivo^(61)^ and checked for word frequency. Final search strings included the search terms ‘(report name)’, ‘meat’, ‘livestock’ and ‘(organisation name)’. A detailed explanation of the search, including a search diary, can be found in Supplemental Text 2. Articles were included in the analysis if the relevant report was mentioned within the article, and the general theme of the article was on meat and/or recommendations for RPM reduction.

### Thematic analysis

Articles were extracted in two steps. First, basic data were entered onto an Excel spreadsheet, including article characteristics (authors, year, title and publisher), the type of article (editorial, news and opinion) and overall article sentiment. Articles were categorised as ‘pro-meat’, ‘neutral’ or ‘pro-reduction’, and then a randomised sample (20 % of total) was independently categorised using the same criteria. Categorisation was conducted by calculating the ratio of supportive/neutral/oppositional sentences to RPM reduction, with the final assessment given to the majority. Inter-reliability between authors was calculated at 90 %. Where category agreement was not reached, team discussions occurred to resolve discrepancies. Articles were then read in entirety and coded in NVivo^(61)^, guided by the framework outlined above. Informed by the theory, an initial coding schema was developed to code the articles (Table 1), and an iterative process was then applied to facilitate adding/modifying codes throughout the analysis, as well as to prevent any constraint from the initial schema^(62)^. Consistent with interpretive research, this involved constant comparison and reflection/refinement of emerging themes by engaging with theory, coded data and discussions with the investigative team^(62,63)^. The authors continually discussed findings to achieve consensus in interpretation. A final list of established themes was agreed upon. These are listed in Supplemental Text 3.

**Table 1:** Coding schema used to guide framing analysis

Key features	Coding prompts
Causation	Is RPM identified as a problem? Who/what is the cause of excessive meat production and consumption?
Harms and benefits	What are the harms of excessive RPM production? What are the benefits? Who is affected by excessive meat production and consumption?
Responsibility	Who is responsible for excessive RPM production/consumption? Whose responsibility is it to address it?
Solutions	What approaches are considered? Which are emphasised over others? What issues are included and excluded? What solutions are opposed?
Actors	Which actor viewpoints are included? How do actors frame other actors in relation to RPM and RPM reduction?

## Results

A total of 150 news media articles were included in the analysis. Articles were primarily news reporting, with 119 news media articles in contrast to 28 opinion or commentary articles. Articles published in response to each report varied in number, with twenty-three articles related to the FAO report, thirty-nine articles related to the WHO report, forty-eight articles related to the EAT-Lancet report and forty articles related to the IPCC report. Across these, sixty-six articles were published by outlets from the United Kingdom, thirty-three from the USA, thirty-three from Australia and seventeen from New Zealand. For ease of reference, the articles have been numbered 1 to 150 (listed in Supplemental Text 3) and are referred to these numbers in the results sections below.

In terms of overall article sentiment, sixty-one articles were categorised as ‘pro-meat’, fifty-three as ‘pro-reduction’ and thirty-two as ‘neutral’. Two articles were categorised as ‘Anti-EAT’ – as the articles themselves were more focused on discrediting the EAT Foundation, rather than RPM reduction itself.

Articles reported on an array of interest groups’ and experts’ responses to the reports, including: academics from health, environment and economics; policymakers; organisational representatives (including the report organisations and non-governmental organisations (NGO)); industry and industry representatives (such as farmers unions); and nutritionists. Views of the article authors were also reflected. Even in articles that were presented as journalistic reporting, many explicitly put forward an editorial opinion of their own in addition to reporting facts and the opinions of stakeholders. Articles that presented mostly quotes from health and environmental academics, organisational representatives and nutritionists tended to skew ‘pro-reduction’, whereas articles that featured predominantly meat industry representatives and economic experts tended to skew ‘pro-meat’.

### The complexity of red and processed meat and its associated health and environmental harms

The perceived complexity of an issue can influence the degree to which it is considered tractable for human intervention^(64)^. Issues that are seen to have ambiguous or complex causes are often less likely to become salient in public discourse and ascend to government policy agendas. Uncertainty over who – or what – is responsible for causing a problem can also lead to ambiguity as to how the problem should be resolved^(64)^. Many actors emphasised the complexity of the issue in their framings, disputing the necessity for any focused policy attention.

Many ‘pro-meat’ articles highlighted the complex relationship between diet, nutrition and good health and thus stressed that proposing RPM reduction was overly simplistic. Many ‘pro-meat’ articles also suggested that the espoused RPM harms lacked scientific consensus (see articles 1, 18, 31, 89 and 140), and thus calls for RPM reduction were presented as unfounded, and in some cases, irresponsible. These views were mostly put forward by industry representatives, for example:

I am very surprised by [WHO]’s strong conclusion on categorising processed meat as ‘definitely’ and red meat as being ‘probably’ carcinogenic to humans given the lack of consensus within the scientific community and the very weak evidence regarding the causal relationship between red meat and cancer. (Meat industry representative; responding to WHO report)

Some ‘pro-meat’ articles also used superlatives and synecdoches to frame the health risks as not only overstated, but farcical (articles 1, 36, 39, 40, 59, 87, 144);

[Editorial voice] ‘We should remember that if we ate processed meat less often, any health risk would be minor anyway, perhaps comparable to breathing in some of the carcinogenic smoke at a vegan summer barbecue.’ (Responding to EAT-Lancet report)

This same frame of complexity was also employed by industry representative groups in relation to environmental harms. Quotes were employed to draw attention to the variety of farming methods and the varying degree in which it contributes to environmental outcomes – for example, pastoral *v*. intensive agriculture.

[Industry organisation] ‘The report is global and doesn’t specifically refer to the Australian industry, which is a world leader in sustainability. Secondly, most of the Australian continent is unsuitable for cropping, making grass-based grazing … the most sustainable way to produce protein in this country’. (Responding to IPCC report)

### Attributing blame for excessive red and processed meat consumption

The media plays a prominent role in agenda-setting, particularly in assigning responsibility for creating or contributing to a given problem. Responsibility can be attributed to individuals and personal choice (and therefore not in the scope of government response) or as a matter of public concern and thus open to a wider distribution of accountability^(65)^. In contrast, the culpability of larger organisations such as government or large-scale corporations can often be unheeded. How much attention is paid to the positioning of responsibility can sway public perception of the best way forward^(65)^.

In most articles, excessive RPM consumption was attributed to individual dietary choices, and thus attenuating the associated harms was solely the responsibility of individual consumers. This view was reflected mostly in ‘pro-meat’ articles, but also by some ‘pro-reduction’ articles (articles 2, 22, 28, 49, 78 and 148). For example, a frame of ‘human consumption excess leading to crisis’ was depicted by several environmental activists.

[Opinion author] The article on the [FAO report] fails to mention that the cattle industry, like the seafood and poultry industries, exists primarily to satisfy the developed world’s insatiable demand for meat… If we overcame our meat-eating habits it would provide everybody with a cleaner environment and a more sustainable future. (Responding to FAO report)

Many ‘pro-reduction’ articles labelled meat eating as a ‘lifestyle choice’ and encouraged readers to become more conscious consumers. Despite this, it was acknowledged that many consumers are not aware of the environmental impact of different foods, partly due to the conflicting messages sent by academia, the media and the government. Several academics and environmental activists were cited positioning the meat industry and their lobbyists as active players in swaying health and climate policy towards corporate interest. Claims of industry-funded science to ‘make the sector look good’ were raised in one ‘pro-reduction’ article (article 21).

Few articles mentioned the role of existing government policies in contributing to and perpetuating high levels of RPM production. One ‘neutral’ article quoted a New Zealand Health Minister acknowledging that the IPCC report had brought to light a failure in public policy on land management and environmental conservation, causing and exacerbating the current climate crisis (article 13). Economic incentives such as agricultural subsidies by governments were noted to be a major obstruction to RPM reduction, creating industry dependence on government (articles 14, 112).

In response to the FAO and IPCC Reports, several ‘pro-meat’ articles attempted to discredit movements towards RPM reduction entirely, minimising the role of agriculture in contributing to the climate crisis relative to other industrial sectors such as fossil fuels (articles 1, 41, 127 and 135). These were promoted mostly by industry representative organisations, as well as some nutrition experts serving as advisors to meat industry bodies. A small number further suggested promoting RPM reduction was backed by other competing industries, such as the UK Soil Association or novel protein corporations like Beyond Meat (articles 5, 72 and 141). This view alluded to the sizeable revenue to be gained by the meat substitute market if RPM consumption were to be reduced.

### Framing solutions to the problems of red and processed meat

How solutions to a given problem are identified and interpreted by policymakers can depend heavily on how they align with wider social values and how the problem itself has been framed in the first place. For example, a ‘personal responsibility’ framing of obesity supports the promotion of more education-oriented policies, such as food labelling or community education^(66)^. Frames can reflect wider societal goals and aspirations, from economic prosperity to social justice to community health and wellbeing^(67)^. As the previous sections demonstrated, a reluctance in accepting the problems associated with high RPM consumption and production meant that solutions were presented in either vague or ambiguous ways, and in some cases, were evaluated with cynicism.

In the articles that accepted the conclusions of the reports and acknowledged health and/or sustainability harms associated with RPM, a host of different solutions were suggested. These solutions included taxing meat, removing agricultural subsidies, creating alternative choices (such as novel protein foods) and improving farming technology to make it less emissions intensive. Except for articles including views from health and environmental academics (articles 23 and 45), most solutions were framed as isolated stand-alone policy actions rather than systemically driven changes targeting different aspects of food systems.

Many industry representatives positioned industry as integral to the solution, without which, attenuating the harms of RPM could not be achieved. Given the role that pastoral grazing-style agriculture can play in sequestering carbon from the atmosphere and preserving some vegetation^(68)^, a large number of meat industry representatives – particularly those in New Zealand and UK – asserted they were well positioned to address some of the environmental concerns (articles 8, 13, 26, 76, 115, 127 and 150).

Despite the solutions being presented as stand-alone interventions in most articles, both ‘pro-meat’ and ‘pro-reduction’ articles mostly agreed that there was no ‘silver bullet’ solution that could be applied globally. Some health academics stressed the importance of not generalising meat reduction recommendations (article 93), as populations in low- and middle-income countries that face the double burden of malnutrition^(69)^ depend on animal-source foods for a nutritionally adequate diet. Furthermore, sustainability academics, as well as farming representatives, argued that not all forms of ruminant farming are alike and some pose less of a risk to sustainability (article 30).

[Industry representative] ‘It says that New Zealand agriculture is getting a lot of things right and is leading the field in terms of finding solutions to achieve better environmental outcomes,’ he said. ‘The report says that New Zealand has become one of the most efficient and environmentally benign ruminant livestock industries’. (Responding to FAO report)

Despite the acknowledgement of this complexity, RPM reduction was primarily framed across most articles as a ‘one size fits all’ policy approach using extreme or indiscriminate terms and thus weighed against the potentially stark consequences to society and loss of civil liberty.

A small number of ‘pro-meat’ articles were sceptical of the effectiveness of some of the proposed actions to reduce RPM in addressing the harms associated with RPM. In some cases, it was represented as not only ineffective, but inadvertently harmful.

[Editorial voice] ‘If we were to tax red meat, many people would switch to more poultry, which is almost always reared on feed, adding to our burden on the planet’. (Responding to EAT-Lancet report)

### Framing the risks of meat and meat reduction

Evaluating the risks associated with a problem, as well those associated with potential solutions is a key component of policymaking. Analysing risks and benefits help determine whether a given solution may be successful in producing the desired outcomes. But *who* benefits, and who is at risk for harm from these outcomes is open to interpretation. Frames that emphasise one viewpoint over another in relation to a policy issue shape and influence the way in which ‘success’, that is, how to overcome that issue, is understood and defined^(45)^.

Proposed actions for RPM reduction were evaluated differently depending on the categorisation of the articles (either ‘pro-meat’ or ‘pro-reduction’). ‘Pro-reduction’ articles largely framed these in a positive light, highlighting the benefits of reduced GHG emissions and lessened chronic health burden on the population. ‘Pro-meat’ articles, however, tended to focus on risks associated with meat reduction. These included (in order of frequency) restrictions on individual liberties and enjoyment (twenty-six mentions), destroying the meat industry and farmers (nineteen mentions), destroying traditions (fifteen mentions), risks to human health from a meat-free diet (twelve mentions), risks to already vulnerable people (seven mentions), the unnatural or undesirable nature of novel proteins (like plant-based imitation meats) (three mentions), risk of economic losses (three mentions) and the fact that other countries would continue to produce RPM, thus nullifying any environmental gains made at home (one mention).

The restriction of choice and the destroying of cultural traditions were repeatedly invoked and largely framed as disproportionately high risk compared to the aforementioned ‘unfounded’ or exaggerated health and/or environmental harms of RPM. This infringement on choice was presented as a means for social control, mostly by an anonymous elite or power-hungry government.

[Opinion author] Frankly I am sick and tired of being lectured to regarding what I should eat, drink and drive etc. … I have no intention of living on a plant-based diet and will continue to enjoy meat as I always have done. (Responding to IPCC report)

### Rhetorical devices used to frame meat reduction

An assortment of rhetorical devices were used to further emphasise frames or ‘to make something look more like one thing than another’ (^(70)^, page 382). Table 2 describes prominent rhetorical devices used across the articles.

**Table 2:** Key rhetorical devices used in the articles

Rhetorical device*	Example frame	Example quote
Metaphor	War on meat	The devil is a shapeshifter … he takes the form of demonic foods. In response, the armies of the righteous have already waged war on sugar, and now red meat is in their sights. (article 1)
Stories of decline	Destroying cultural and traditional values	‘Isn’t it remarkable’, wrote [academics], ‘how meat, symbolising health and vitality since millennia, is now often depicted as detrimental to our bodies, the animals and the planet? Why exactly is the minoritarian discourse of vegetarianism and veganism currently all over the media?’ (article 5)
Story of helplessness and control	Reducing meat hurts poor people	Many people are already struggling to make ends meet, … so how is putting a [tax] on [meat] going to solve anything? It will be interesting to see … if all those following vegan and vegetarian diets do not succumb to osteoporosis amongst other illnesses because of lack of proper nourishment. (article 130)
Symbol	Systemic meat reduction restricts personal liberties	On the downside, this [EAT-Lancet] diet would not exactly be a riot of flavour. But on the upside, your entire meat bill for future Australia Day barbecues could be covered by spare change. (article 39)
Using numbers to tell a story	Human consumption excess leading to crisis	With increased prosperity, people are consuming more meat and dairy products every year. Global meat production is projected to more than double from 229 million tonnes in 1999/2001 to 465 million tonnes in 2050, while milk output is set to climb from 580 to 1043 million tonnes. (article 64)

*Categories are sourced from ref. [70].

### The ‘Vegan Agenda’ and the fight for control

A strong feature of all articles was the framing of other actors in relation to meat reduction. This was generally applied as a means of discrediting or delegitimising opponents to support their own either ‘pro-reduction’ or ‘pro-meat’ frame. For example, industry representatives or local farmers and butchers frequently questioned the veracity of the reports, suggesting that scientists had generalised or made inappropriate recommendations based on correlative, not causative evidence (articles 31, 57 and 138). This was underscored with an assertion that scientists were conducting themselves in a ‘dubious’ or unreliable manner.

The media were also specifically blamed by industry and ‘pro-meat’ advocates, as reported in several articles, for perpetuating and misleading the public on the findings of each report. The media were labelled as ‘hyperbolic’ and accused of being selective of the various reports to further ‘anti-meat’ goals, tarnishing the reputation of the meat industry (articles 88, 96, 117, 125, 135 and 144).

Some ‘pro-meat’ framings portrayed opponents as ideologically charged. Meat reduction was often positioned as part of a wider conspiracy to not just reduce meat production and consumption but rather to turn the world vegan (meaning no meat or animal products to be produced and consumed at all). This was frequently referred to as the ‘Vegan Agenda’. In some articles, the existence of the ‘Vegan Agenda’ was supported by proffering examples of UK government ministers who were themselves vegan and who supported the findings of the reports.

[Economic expert] ‘They are making no secret of their desire to tax and ban their way to a near vegan diet for the world’s population. Their desire to limit people to a tenth of a sausage a day leaves us in no doubt we are dealing with fanatics. They say they want to save the planet but it is not clear which planet are they on’. (Responding to EAT-Lancet report)

Those that did not go so far as to suggest that a conspiratorial agenda was evident in the reports themselves claimed that environmental activists were co-opting the more measured scientific recommendations in the reports to advance vegetarianism and veganism.

[Industry organisation] Unfortunately, … the report has been hijacked by those with an anti-meat agenda (read: vegan activists) as ‘proof’ that we all need to switch to a plant-based diet to save the planet. (Responding to IPCC report)

The ‘Vegan Agenda’ frame was associated with blame and guilt for those who choose to eat meat, and public shaming towards those who could not afford vegan meat alternatives. Thus, a power hierarchy was depicted between ‘the people’ and ‘the elite’.

[Editorial voice] Is it possible that a combination of well-meaning philanthropists and large agricultural concerns have united to exploit health fears for financial gain, while neglecting the nutritional shortcomings in their recommendations? (Responding to conspirative concerns of the EAT-Lancet report)

Governments were also vulnerable to being framed in both ‘pro-meat’ and ‘pro-reduction’ stories. Industry actors accused governments of politicising evidence, as part of a wider desire to control citizens in a ‘nanny-state’ setup. Conversely, pro-reduction academics accused governments of being ‘in bed with industry’ and prioritising corporate interest over public and planetary health. This framing was also applied by academics towards industry, portraying the meat sector as short-sighted in the wider environmental crisis and as malevolent and solely self-interested.

Finally, the media also pointed fingers at consumers. Consumers were frequently framed by journalists as complacent, ignorant of food origins and contradictory in their stated desires (e.g. wanting food that was both environmentally friendly AND cheap).

## Discussion

This study aimed to understand how RPM reduction as a strategy for achieving healthy and sustainable food systems is interpreted and portrayed (i.e. framed) in news media by different groups with an interest in RPM in the USA, UK, Australia and New Zealand.

This study found that news media articles contained viewpoints from actors spanning diverse sectors, including the meat industry, academics, policymakers, NGO, as well as the media itself. The public and planetary health harms associated with RPM were frequently disputed, mostly by meat industry representatives, primarily by framing the issue as too complex and ambiguous, with scientific evidence lacking or erroneous. Highlighting the complexities of food-health relationships by keeping other, more general drivers of poor health and sustainability in focus, helps to assuage scrutiny over specific harmful commodities^(71)^. This is a consistently utilised frame in relation to other health issues connected with risk commodities, such as obesity and sugar-sweetened beverages^(72,73)^. Multinational food and beverage companies such as Coca Cola publicly disputes (as well as funds the production of evidence to contradict) the scientific links between overconsumption of their products and risk for obesity and chronic disease by drawing on other contributing factors such as sedentary lifestyles^(74)^, so that policy solutions that directly impact their production are less politically favourable. In the case of RPM, the definition of what constitutes ‘red meat’ and ‘processed meat’ enables this complexity, as well as levels of consumption and which specific forms of production are ascribed to particular environmental harms. Many articles did not differentiate between meat, red meat and processed meat in their content, nor make any distinctions within these arguably broad categories. Obscuring the relationship between food and health or sustainability allows for the efficacy of specific policy actions to be contested. Moreover, by simplifying the recommendations and their purported intended outcomes (e.g. ‘reducing meat consumption means everyone will be vegan’) support for their implementation wanes.

Articles including viewpoints of health and environmental academics showed support for the findings of each report, as well as for recommendations to reduce overall RPM intake. This aligns with a social justice-based framing, where net benefits of reduction are positioned within the context of social amelioration. For example, RPM reduction was framed as having a positive impact on population health, a key component to reducing GHG emissions and a means of combatting industry tactics designed to increase consumption. These ‘big picture’ frames are consistent with other ‘risk commodities’ such as alcohol, tobacco or sugar^(75)^.

The reporting of viewpoints of policymakers and government spokespeople was sparse across the articles, perhaps representative of the low political salience this issue currently holds in these four countries.

The views of the authoring journalists and media outlets were both overtly and covertly evident in each article. Contextualising news media as actors in their own right, embedded as active members in the policy process has become increasingly important, given their power in determining not only *which* items are sent into the public agenda, but *how* ^(65)^. For example, the narratives employed by news media to describe and explain issues and proposed solutions can determine the context in which public opinion, and therefore policy agendas, are defined.

### Polarisation and identity: Vegans *v*. meat lovers

The four reports used as reference points for this analysis are highly technical and present complex evidence, yet very few of the frames utilised in the included articles related to evidence. In contrast, almost all frames were value-based or interest-driven. Presenting the issue this way results in decontextualised discussions and an oversimplified discourse, where contrasting voices are given equal attention, and thus command equal authority – regardless of their consistency with the evidence. This is reflective of a broader trend in both news and social media – the growing paucity of nuance and complexity in discussions of contemporary social, economic and environmental issues^(76,77)^. Effective and valuable policymaking to attenuate the harms associated with RPM depend on the capacity for nuanced and truthful discussions in public fora.

Meat reduction framing in these articles is reflective of these polarising tendencies in the media. There appeared a stark binary of ‘Vegan Agenda’ *v*. ‘meat lovers’, and these descriptions carried several assumed and stereotyped interpretations. The ‘Vegan Agenda’ was portrayed as a movement designed to entirely remove the option of meat consumption and mobilise a vegan diet worldwide. This ties closely with Deborah Stone’s concept of ‘characterisation’^(70)^, using synecdoches to frame issues with reference to a wider social group (the ‘hero’) and its competitors (the ‘villains’). In this context, the ‘Vegan Agenda’ is used as a symbol that represents a threat to tradition and enjoyment of RPM. The idea of the ‘Vegan Agenda’ is embedded in neoliberal ideology, which emphasises the importance of individual choice and free will^(78)^. It also links closely with the aforementioned Carnism ideology, which reinforces the ‘natural’ and ‘normal’ place of meat in society^(21)^. It implies that vegans want to impose their personal lifestyle choice on the individual choices of others, which should be considered inviolable. This is a commonly deployed framing, encapsulated under ‘market justice’ by Weishaar *et al*.^(79)^ – i.e. regulation by the state takes away personal and commercial freedoms. However, it is important to note that historically there have been vegan movements that have implored the necessity of universal vegan diet adoption^(80,81)^, primarily driven from animal-welfare considerations. How these movements have been conflated with more measured appeals for sustainable levels of meat consumption is a question for future research, particularly in relation to the use of the term ‘meat reduction’ by these various groups.

Conversely, it was evident that many producers and their representative organisations perceived their industry to be painted with one broad stroke. Many articles featured stories of the sector aiming to distinguish the varying ways in which RPM is produced, both at the farming and processing levels, and thus the variable impacts on health and environmental outcomes. It is also possible that media articles relied on the voices of individual graziers and their representative groups to better connect with romanticised notions of traditional food production and rural values connected with their lived experience running a farm. In comparison, large-scale multinational meat processing companies are less positioned to tap into this public sentiment, particularly in countries like the UK, the USA, Australia and New Zealand, the latter three with a British colonial history, where pastoral expansion and British food culture have integrated into national identity^(82)^. Voices from large-scale industrial meat processers such as JBS or Tyson Foods (which operate in all four countries to various degrees) were noticeably absent from the data, despite their significant contributions to these public and planetary harms relative to smaller-scale and less-intensive producers. However, whilst there are ecologically sensitive opportunities in some forms of livestock farming, evidence suggests that even if these technical improvements were universally incorporated, they would be unable to simultaneously meet both goals of attenuating environmental harms and sustaining current levels of meat consumption^(83)^.

The binary between ‘vegans’ and ‘meat lovers’ means that the problem and corresponding proposed solutions are polarised and emotive, and thus the capacity to respond to evidence becomes shrouded by a more value-driven debate. This also extends to ostensibly ‘catch all’ terms such as ‘plant-based’, which has increasingly become associated with veganism in public fora, despite widespread understanding in public health nutrition academic fora as a dietary pattern consisting mostly of fruits, vegetables, nuts, seeds, oils, whole grains, legumes, and a small amount of eggs, dairy and meat if desired^(84)^. These broad generalisations contribute further to a wider social phenomenon of identity and opposition; one is either ‘pro-meat’ or ‘anti-meat’, with seemingly nothing in between. The consequences result in a stark and emotionally driven flow of information, where individuals are unable to empathise with other parties and remain unmoved by evidence. Furthermore, as the power of ‘Big Tech’ comes to light, personalising algorithms give rise to disparate sets of evidence and information being provided to the general public^(76)^. As such, public debate is being conducted in separate spheres of knowledge, with no understanding of (or desire to understand) the viewpoints of the other side^(76)^.

### Capitalising on polarised debates by industry

Challenging evidence and discrediting scientific data is a time-old technique from the corporate playbook, particularly in the realm of climate and environmental policymaking efforts. By intentionally disseminating doubt over the consensus of evidence, the general public is less likely to support public policies that are reliant on that evidence^(85)^. Corporations in other harmful industries have used this strategy to combat growing awareness of their harms, for example, cigarette smoking and cancer^(86)^, human impact on climate change^(87)^, and sugary drinks and obesity^(88)^. By employing frames to sow doubt about the strength of evidence on the harms of RPM, the industry can work to diminish their contributions and mitigate public support for any policies that may create an unfavourable or unprofitable environment for their products. Additionally, they may extend this by coordinating or providing grants or financial assistance towards academic research to shape favourable knowledge environments^(89)^.

In particular, large-scale meat processing corporations such as JBS or Tyson Foods, and ancillary animal feed corporations such as Cargill, contribute a substantial proportion of the health and environmental harms associated with RPM^(90)^. Despite this, no representatives from these corporations were included in any of the news media articles. In contrast, meat industry perspectives largely emanated from local farmers or peak industry bodies. Literature on corporate tactics to engage and shape policy has grown over the last decades; however, how companies lobby through representative and trade associations specifically lacks analysis. Arguably, using third-party rebuttals to respond and contend with public discourse allows large companies to maintain their public image and avoid a ‘David and Goliath’-esque depiction in public debate^(91)^. Consistent with other harmful industries, large transnational food corporations tend to vicariously work through industry associations, which allow them to lobby for their interests without being seen as directly responsible^(92)^.

Other industries have also managed to gain public social licence during this time. Whilst the data collected for this analysis were drawn from specific points in time (2006, 2015 and 2019), the public discourse surrounding meat reduction has continued to evolve. At the time of writing, there has been a significant rise in the popularity of novel proteins produced by companies such as Beyond Burger and Impossible Foods, which have capitalised on the rhetoric surrounding plant-based diets and RPM reduction, with some companies purporting to work towards the aim of completely meatless diets – despite their own products having potential human health and environmental risks^(93)^.

### Strengths and limitations

This study’s investigation of the framing of RPM narratives in the media addresses a gap in the literature on a topic that has become increasingly contentious in the healthy and sustainable food systems discourse. It helps explain how policy is being influenced now and provides insights as to how policymaking might be improved in the future. The following limitations of the method should be applied to the interpretation of the results. Firstly, this study focused on only four countries that specifically have high RPM production and consumption, and thus the findings cannot necessarily be generalised to other countries. Secondly, RPM reduction is an ongoing topic of media discourse, particularly in the context of novel proteins and the worsening climate crisis. Our findings only relate to a particular point in time and agricultural practices are likely to evolve in the future. Further, the ProQuest database may not have captured all relevant data for analysis: in particular, despite the sizeable presence of the meat industry in the USA, articles from that country were underrepresented. Finally, the political leanings of the news media outlets were not considered, which may have elucidated which frames were being utilised with different audiences.

## Conclusion

The findings of this study indicate that the challenge of RPM reduction in news media is understood and interpreted in a variety of ways; from being a means to reduce carbon emissions and prevent cancer, to being a radical attempt to control individual diets and choices. The study identified mostly polarising and emotive frames being employed by actors, including the meat industry. To avoid being framed as extreme and prescriptive, recommendations for RPM reduction must instead be framed with nuance and context; as a significant reduction in *average* production and consumption – rather than a universal and equal reduction for all. Defining what is meant by red meat and processed meat is also important, particularly in the rising acknowledgement of ultra-processed meats such as chicken nuggets or hot dog meat. Recognition that some consume too much for planetary boundaries and health (such as those in high-income countries), but that also some (especially in low- and middle-income countries) may benefit from sustained or increased consumption, is required and advocating for universal diet adoption globally is not appropriate. The harms of RPM are a complex issue that need complex and contextual solutions – it cannot be ‘all or nothing’ binary narratives. Traditional news media have long been criticised for oversimplifying complex policy problems, but this phenomenon has been further amplified with online media. A topic for further research is how this can be reconciled with the nature of ‘click-bait’ media, as well as selective algorithms that predetermine what evidence is presented to whom. The role of media itself as a persuasive actor is increasingly important in these contexts.

The findings show that polarised frames of RPM reduction have led to a binary conflict between pro- and anti-meat reduction actors. This division may diminish the extent to which political leaders will prioritise this in policy agendas and poses a challenge for public health messaging to ensure messages are both (1) nuanced and context-dependent and (2) consistent.

## Supplementary material

For supplementary material accompanying this paper visit https://doi.org/10.1017/S1368980021004092. click here to view supplementary material
