# Supplementary material for: Who has a beef with reducing red and processed meat consumption? A media framing analysis
Source: Public Health Nutr. 2021 Sep 30;25(3):578–90. doi: 10.1017/S1368980021004092 (PMC9991568; doi:10.1017/S1368980021004092)
Supplement: Supplementary file 1 [file S1368980021004092sup.zip › S1368980021004092sup002.docx]

**Supplementary Text 1: Search diary**

***Database searches***

We initially undertook iterative searches of the ProQuest databases as well as a sample of newspaper webpages, selected for their relevance and comprehensiveness after consultation with the Deakin University research librarian liaison trained in systematic search. Search strings were revised through preliminary searches of each database and assessed for specificity and sensitivity. The results of each iteration were reviewed for relevance and comprehensiveness until an optimal search string was identified.

| **Database** | **Date (by)** | **Search string** | **Limits applied** | **No. hits – prelim. search** | **No. hits – final search** | **Date exported on** |
| --- | --- | --- | --- | --- | --- | --- |
| ProQuest (International Newsstream) | 6.07.20 | ("EAT-Lancet" OR "EAT Lancet" OR “Lancet”) and (“meat” OR “reduc*” OR "Red" OR “Processed” OR “agricultur*” OR “animal source*” OR “diet*”) | Language: English  Type: Newspaper  Source: US/Australia/NZ/UK Dates: Dec 2018 - June 2019 | 5,822 | 385 | 6.07.2020 |
| ProQuest (International Newsstream) | 6.07.20 | ("FAO" OR "Livestock* Long Shadow" OR "Food and agriculture organi*") AND (“meat” OR “reduc*” OR "Red" OR “Processed” OR “livestock” OR “production”) | Language: English  Type: Newspaper  Source: US/Australia/NZ/UK Dates: Sept 2006 – April 2007 | 417 |  |  |
| ProQuest (International Newsstream) | 6.07.20 | ("FAO" OR "Livestock* Long Shadow" OR "Food and agriculture organi*") AND (“meat” OR “reduc*” OR "Red" OR “Processed” OR “livestock” OR “production”) | Language: English  Type: Newspaper  Source: US/Australia/NZ/UK Dates: Oct 2006 – Feb 2007 | 285 |  | 6.07.2020 |
| ProQuest (International Newsstream) | 7.07.20 | ("IPCC" OR "International Panel on Climate Change" OR "Climate and land use" OR "Special report") AND (“meat” OR “agricultur*” OR “production” OR “ruminant” OR “livestock” OR “food”) | Language: English  Source: US/Australia/NZ/UK  Type: Newspaper Dates: June 2019 – November 2019 | 209 |  | 7.07.2020 |
| ProQuest (International Newsstream) | 7.07.20 | ("WHO" OR "World Health Organi*" OR "Monographs" OR "International Cancer Research" OR “IARC”) AND (“meat” OR “reduc*” OR "Red" OR “Processed” OR “beef” OR “pork” OR “diet*”) | Language: English  Source: US/Australia/NZ/UK  Type: Newspaper Dates: August 2015- February 2016 | 4632 | 303 | 7.07.2020 |
