# Supplementary material for: Who has a beef with reducing red and processed meat consumption? A media framing analysis
Source: Public Health Nutr. 2021 Sep 30;25(3):578–90. doi: 10.1017/S1368980021004092 (PMC9991568; doi:10.1017/S1368980021004092)
Supplement: Supplementary file 1 [file S1368980021004092sup.zip › S1368980021004092sup003.docx]

| **Report** | **Specific recommendations on meat production and/or consumption** |
| --- | --- |
| *FAO “Livestock’s Long Shadow”* | In the varied contexts of the livestock sector, environmental issues require an integrated approach, combining policy measures and technology changes, within a framework of multiple objectives. The livelihood concerns of hundreds of millions of poor livestock holders, who often engage in livestock production because they have no alternative, must be taken into account. The demands of the emerging middle class, who are consuming growing amounts of meat, milk and eggs, cannot be ignored either. Attempts to curb the booming demand for these products have generally proved ineffective. |
|  | Better policies in the livestock sector are an environmental requirement, and a social and health necessity. |
|  | Neglect may also stem from the strong lobbying influence that livestock producers wield in many countries, particularly developed ones (Leonard, 2006). This affects the political economy of public policy making in the livestock sector in the EU, the USA, Argentina and elsewhere. It is often argued that in the past, livestock lobbies have been able to exert an over-proportional influence on public policies, to protect their interests. An indication of this lobbying power is the persistence of agricultural subsidies, amounting to an average of 32 percent of total farm income in OECD countries, with livestock products (dairy and beef, in particular) regularly figuring among the most heavily subsidized products. |
|  | Livestock sector policies need to address a host of economic, social, environmental and health objectives. In most cases, it will be impossible to design policies that will address all at once and at reasonable costs to government and the people affected. Though important trade-offs exist and compromises need to be made. |
| *IPCC “Land use and climate”* | Further, a number of response options such as increased food productivity, dietary choices and food losses, and waste reduction, can reduce demand for land conversion, thereby potentially freeing land and creating opportunities for enhanced implementation of other response options (high confidence) |
|  | Response options throughout the food system, from production to consumption, including food loss and waste, can be deployed and scaled up to advance adaptation and mitigation (high confidence). The total technical mitigation potential from crop and livestock activities and agroforestry is estimated as 2.3 – 9.6 GtCO2eq yr-1 by 2050 (medium confidence). The total technical mitigation potential of dietary changes is estimated as 0.7 – 8 GtCO2eq yr-1 by 2050 (medium confidence). |
|  | Diversification in the food system (e.g., implementation of integrated production systems, broad-based genetic resources, and diets) can reduce risks from climate change (medium confidence). Balanced diets, featuring plant-based foods, such as those based on coarse grains, legumes, fruits and vegetables, nuts and seeds, and animal-sourced food produced in resilient, sustainable and low-GHG emission systems, present major opportunities for adaptation and mitigation while generating significant co-benefits in terms of human health (high confidence). By 2050, dietary changes could free several million km2 (medium confidence) of land and provide a technical mitigation potential of 0.7 to 8.0 GtCO2eq yr-1, relative to business as usual projections (high confidence). Transitions towards low-GHG emission diets may be influenced by local production practices, technical and financial barriers and associated livelihoods and cultural habits (high confidence). |
|  | Public health policies to improve nutrition, such as increasing the diversity of food sources in public procurement, health insurance, financial incentives, and awareness-raising campaigns, can potentially influence food demand, reduce healthcare costs, contribute to lower GHG emissions and enhance adaptive capacity (high confidence). Influencing demand for food, through promoting diets based on public health guidelines, can enable more sustainable land management and contribute to achieving multiple SDGs (high confidence). |
| *WHO IARC Monographs on the evaluation of carcinogenic risks to humans*  *“Red meat and Processed Meat”* | Consumption of red meat is *probably carcinogenic to humans (Group 2A)*. Consumption of processed meat is *carcinogenic to humans (Group 1)*. |
| *The EAT-Lancet Commission on Healthy Diets from Sustainable Food Systems “Our Food in the Anthropocene”* | Food subgroup: Beef, lamb, pork  Reference diet (g/day): 14  Possible ranges (g/day): 0 to 28 |
|  | A transformation of the global food system must ultimately involve multiple stakeholders, from individual consumers to policy makers and actors along the food value chain, working together toward the shared global goal of healthy and sustainable diets for all. |
|  | Transformation to healthy diets by 2050 will require substantial dietary shifts, including a greater than 50% reduction in global consumption of unhealthy foods such as red meat and sugar, and a greater than 100% increase in the consumption of healthy foods such as nuts, fruits, vegetables and legumes. However, the changes needed differ greatly by region. |
|  | In this Commission, we do not propose a magic global fix to the problems discussed. Instead, the safe operating space for food systems, as defined by this Commission, will require implementation of a variety and multitude of solutions and innovations to achieve healthy diets from sustainable food systems. |
|  | Because animal source foods can have important influences on both human health and environmental sustainability, particular detail will be given to these foods. However, the conclusions of this chapter are based only on health outcomes. Although important, we do not consider food safety (i.e. microbial or other forms of contamination). |
|  | Protein “quality” reflects the amino acid composition of the food source, and animal sources of protein are of “higher quality” than most plant sources. This is particularly important for growth of infants and young children, and possibly in elderly persons who are losing lean mass.52 However, a mix of amino acids that maximally stimulates cell replication and growth may not be optimal throughout most of adult life because more rapid cell replication is a concern for cancer risk. |
|  | In a major review, the 2015 U.S. Dietary Guidelines Advisory Committee concluded that for persons over two years of age, a balanced vegetarian diet can be a healthy eating pattern. In the largest prospective study of vegetarian diets, those following vegan, lacto-ovo, pesco-vegetarian, or semi-vegetarian diets together had a 12% lower overall mortality risk compared to omnivores; the lowest risk was among pesco-vegetarians.55 Using another approach, a plant-based dietary score, giving positive values to the frequency of plant products (especially healthy plant-based foods, but not refined grains) and negative values to animal products, was associated linearly with lower risk of type 2 diabetes and coronary heart disease.56,57 These findings suggest that a shift towards a dietary pattern emphasizing whole grains, fruits, vegetables, nuts, and legumes without necessarily becoming a strict vegan, will be beneficial. |
|  | In the 1960s, when Greek men living in Crete had very low rates of coronary heart disease and overall mortality, their average intake of red meat and poultry combined was 35 grams/day. |
|  | In a pooled analysis of Asian cohort studies, poultry and red meat consumption (mainly pork) was associated with lower all-cause mortality.75 The discrepancies between this analysis and those from Europe and North America may be explained in part by the fact that Asian populations eat much less meat. Also noted by the authors, the findings could be due to confounding factors because meat may be more available to individuals of higher socioeconomic status, who also have better overall health. Most importantly, because many of these countries have only recently become affluent, the current levels of red meat intake do not reflect long-term intakes, like for smoking, many decades are likely needed to experience the full health consequences of high consumption. Among Chinese living in Singapore, which has been relatively affluent for several decades, red meat consumption has been associated with risk of type 2 diabetes,76 consistent with the overall literature on this relationship. |
|  | Because intake of red meat is not essential and appears to be linearly related to higher total mortality and risks of other health outcomes in populations that have consumed it for many years, the optimal intake may be zero, especially if replaced by plant sources of protein. Because precision about risk at low intakes is limited, we conclude that a low range of intake, 0 to approximately 28 grams/day, is desirable and have used a midpoint of 14 g/day for the reference diet. As consumption of poultry, compared to red meat, has been associated with better health outcomes, we have used a range of 0 to approximately 58g/day and a midpoint of 29 grams per day for the reference diet. |
